# Benign and Malignant Peripheral Nerve Sheath Tumors of the Oral Cavity: Two-Case Series Emphasizing Diagnostic Challenges

**DOI:** 10.3390/diagnostics16132039

**Published:** 2026-06-30

**Authors:** Evgeniy Aleksiev, Dimitar Kosturkov, Tihomir Dikov, Vesela Ivanova, Zornitsa Mihaylova

**Affiliations:** 1 Research Institute of Innovative Medical Science, Medical University—Sofia, 1431 Sofia, Bulgaria; 2 Department of Dental, Oral and Maxillofacial Surgery, Faculty of Dental Medicine, Medical University—Sofia, 1431 Sofia, Bulgaria; 3 Department of Conservative Dentistry, Faculty of Dental Medicine, Medical University—Sofia, 1431 Sofia, Bulgaria; 4 Department of General and Clinical Pathology, Faculty of Medicine, Medical University—Sofia, 1431 Sofia, Bulgaria; 5 Alexandrovska University Hospital, 1431 Sofia, Bulgaria

**Keywords:** malignant peripheral nerve sheath tumor, schwannoma, oral cavity tumors, lower lip neoplasm, tongue lesion, peripheral nerve sheath tumor, malignant transformation

## Abstract

**Background:** Peripheral nerve sheath tumors of the oral cavity are rare and encompass both benign and malignant entities. Differentiating between these lesions remains challenging due to overlapping clinical and histopathological characteristics. **Case presentation:** We present two cases illustrating the biological spectrum of peripheral nerve sheath tumors in the oral cavity. The first case involves a 76-year-old male with a recurrent lower lip lesion initially diagnosed as benign, which progressed to a high-grade malignant peripheral nerve sheath tumor (MPNST). The second case describes a 20-year-old male presenting with a nodular lesion of the tongue, initially suspected to be reactive following trauma, but histologically confirmed as a benign schwannoma. Both patients underwent surgical treatment with favorable immediate postoperative outcomes. **Conclusions:** These cases highlight the diagnostic complexity and heterogeneous behavior of peripheral nerve sheath tumors. Histopathological and immunohistochemical evaluations are essential for definitive diagnosis. Clinicians should maintain a high index of suspicion and consider possible association with NF1 or schwannomatosis, particularly in recurrent or atypical lesions.

## 1. Introduction

Peripheral nerve sheath tumors (PNSTs) are a heterogeneous group of neoplasms arising from Schwann cells, perineural cells, and fibroblasts [1,2]. These tumors include benign entities such as schwannomas and neurofibromas, as well as malignant peripheral nerve sheath tumors (MPNSTs), which represent aggressive soft tissue sarcomas [3,4].

MPNSTs account for approximately 5–10% of all soft tissue sarcomas and are frequently associated with neurofibromatosis type 1 (NF1) [5,6,7]. In contrast, schwannomas are benign, slow-growing tumors with an excellent prognosis following complete excision [8,9].

A substantial proportion of MPNSTs arise in association with hereditary tumor predisposition syndromes, particularly Neurofibromatosis Type 1 (NF1), whereas schwannomas may occur sporadically or in association with schwannomatosis syndromes. Patients with NF1 demonstrate a significantly increased lifetime risk of developing MPNST, most commonly through malignant transformation of pre-existing plexiform neurofibromas [5,6,7,10].

Histopathologically, NF1-associated MPNSTs may demonstrate transition zones between benign plexiform neurofibroma and overt sarcoma, accompanied by increased cellularity, nuclear atypia, mitotic activity, and infiltrative growth. At the molecular level, recurrent alterations involving NF1, TP53, CDKN2A, SUZ12, and EED have been implicated in malignant progression [2,3,6,11]. In contrast, benign schwannomas typically exhibit diffuse S100 and SOX10 positivity, Antoni A/B architecture, and low proliferative activity [8,9,11].

Surgical management of oral PNSTs may also be particularly challenging because of the anatomical complexity of the oral cavity and the need to preserve speech, swallowing, oral competence, and facial aesthetics while achieving complete tumor excision.

In the oral cavity, PNSTs are rare, and their clinical presentation is often nonspecific, which may lead to delayed or incorrect diagnosis [3,12]. Imaging modalities such as CT and MRI may aid in evaluation, but definitive diagnosis relies on histopathological and immunohistochemical assessment [12,13,14].

This case series aims to highlight the diagnostic pitfalls and biological continuum of oral PNSTs, emphasizing the importance of early recognition of malignant features and the limitations of initial histopathological assessment.

We present two cases of PNSTs in the oral cavity, demonstrating contrasting biological behavior, emphasizing diagnostic challenges and clinical implications.

## 2. Case Presentations

Case 1

A 76-year-old male presented with a progressively enlarging mass of the lower lip over approximately 18 months. The lesion developed at the site of a previous surgical excision performed in 2021, when histopathology revealed an intramuscular hemangioma.

Clinical examination showed a firm nodular lesion measuring approximately 25–35 mm, involving the full thickness of the lower lip. Palpation identified additional nodular structures along the course of the left mental nerve, which appeared thickened and cord-like. No cervical lymphadenopathy was detected.

Computed tomography demonstrated a soft tissue mass measuring 37 × 25 × 27 mm, without evidence of bone invasion or regional lymphadenopathy, consistent with previously described imaging features of peripheral nerve sheath tumors [12,13].

An incisional biopsy suggested a peripheral nerve sheath tumor, with differential diagnosis including neurofibroma and schwannoma. The patient subsequently underwent radical surgical excision with reconstruction of the lower lip.

**Figure 1:**
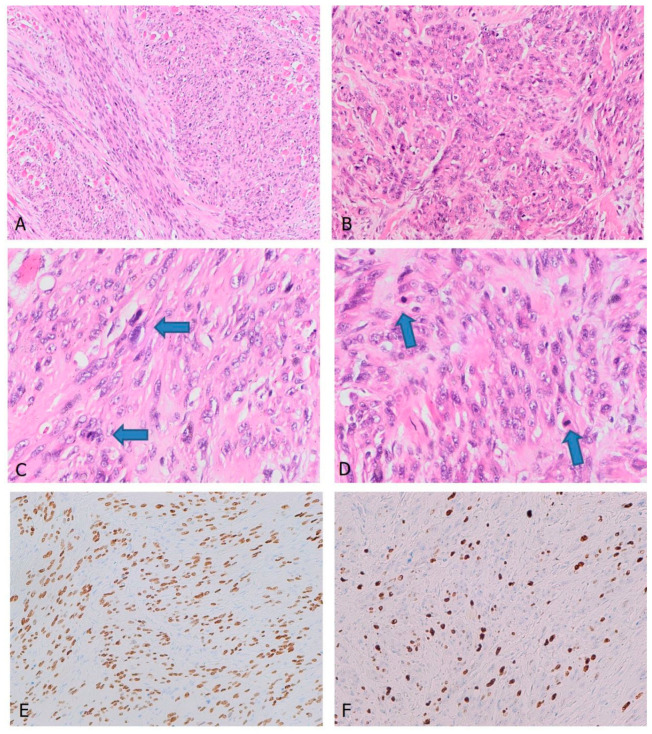
(**A**) Hematoxylin and Eosin (HE) staining, objective ×10; (**B**) HE, objective ×10; (**C**,**D**) HE, objective ×40. Hypercellular tumor tissue, composed of fascicles of spindled cells with noticeable nuclear variability (**C**, arrows) and an increased number of mitotic figures (**D**, arrows). (**E**) SOX10, objective ×20, (**F**) Ki67, objective ×20. Immunohistochemical study disclosed nuclear expression of SOX10 and Ki67-proliferative index averaging 45%.

Histopathological examination (Figure 1) revealed a spindle-cell neoplasm with fascicular architecture, moderate to severe cytological atypia, and high mitotic activity (>10 mitoses/10 HPFs). Immunohistochemistry showed positivity for SOX10 and a Ki-67 proliferation index of up to 45%, consistent with high-grade MPNST. Tumor infiltration along the mental nerve with plexiform architecture suggested malignant transformation, possibly associated with NF1 [5,6]. No confirmed clinical diagnosis of NF1 was documented at presentation, and the patient did not report a known history of hereditary tumor syndromes. No *café-au-lait* macules, multiple cutaneous neurofibromas, or positive family history suggestive of NF1 were documented during clinical evaluation. However, the plexiform growth pattern, neural distribution, and malignant transformation strongly raised suspicion for an underlying NF1-associated lesion. Genetic testing was not available in the present case, representing a limitation of the current report.

**Figure 2:**
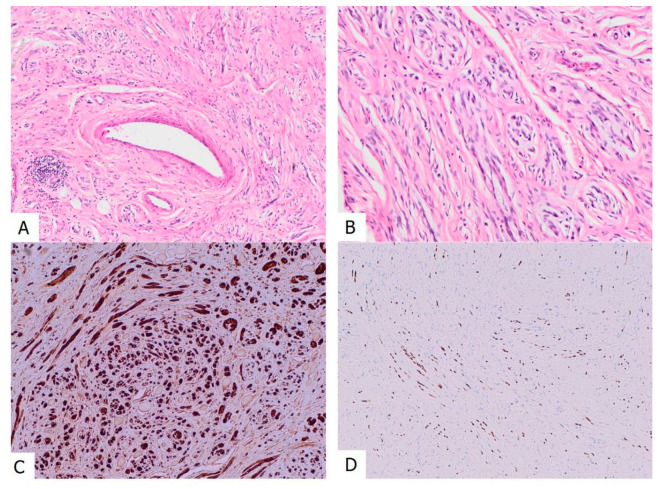
(**A**) Hematoxylin and Eosin (HE) staining, objective ×10; (**B**) HE, objective ×20. Moderately vascularized tumor tissue, generally inconspicuous spindle-shaped cells, devoid of any nuclear atypia. (**C**) S100, objective ×10; (**D**) SOX10, objective ×10. Follow-up immunohistochemical study displaying strong S100-immunopositivity and weak to moderate nuclear expression of SOX10.

The final diagnosis was high-grade malignant peripheral nerve sheath tumor (MPNST), pT2N0M0R0. This prompted retrieval of the archival biopsy tissue dating from 2021 and subsequent revision of the diagnosis. On second look, only few and rare spindled cells were noted, arranged at tiny fascicles and definitely lacking any signs of atypia (Figure 2). These findings, coupled with conspicuous blood vessels hinted at Antoni B areas in possible schwannoma. The tissue was submitted for immunohistochemical stains (S100, SOX10), confirming diagnosis of preexisting benign PNST.

The postoperative course was uneventful, and the patient was referred for oncological follow-up with a surveillance strategy in accordance with current recommendations [15,16].

Case 2

A 20-year-old male presented with a nodular lesion on the left side of the tongue tip, first noticed approximately two months prior to admission. The patient associated the lesion with repeated trauma due to accidental biting.

Initially, the lesion regressed but subsequently recurred and persisted. Clinical examination revealed an exophytic nodular lesion approximately 1 cm in diameter, with a pinkish-whitish surface and focal superficial erosions. The lesion was firm on palpation, with slight induration at its base. No cervical lymphadenopathy was present.

The patient had no significant medical history. Laboratory findings were within normal limits. Differential diagnosis included pyogenic granuloma and benign neoplasm.

Complete surgical excision was performed under local anesthesia with intravenous sedation. The postoperative course was uneventful.

**Figure 3:**
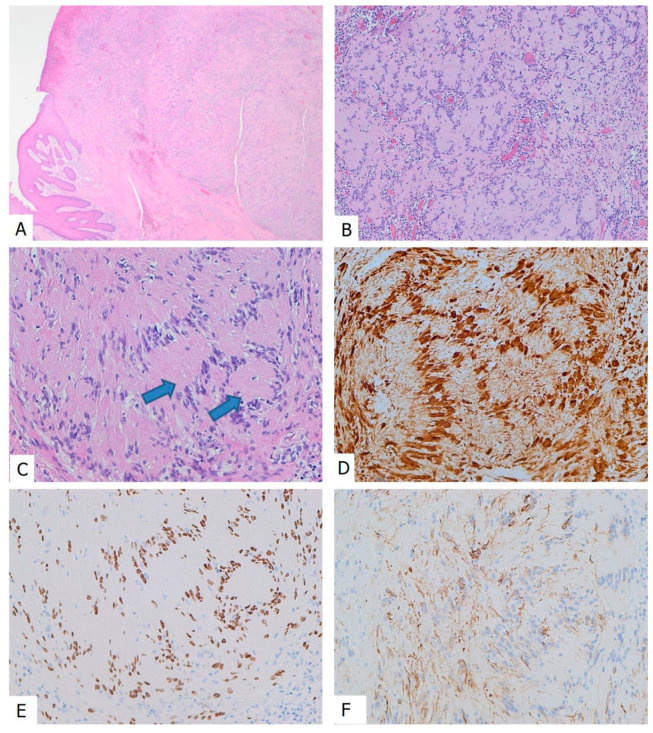
(**A**) Hematoxylin and Eosin (HE) staining, objective ×2; (**B**) HE, objective ×10; (**C**) HE, objective ×20. Ulcerated squamous epithelium with underlying nonspecific mixed inflammatory infiltrate and a piece of tumor tissue. The latter is predominantly composed of solid areas consistent with Antoni A growth pattern with an abundance of Verocay bodies (arrows). Non-significant atypia is noted, as well as mitotic activity. (**D**) S100, objective ×20; (**E**) SOX10, objective ×20; (**F**) GFAP, objective ×20. Immunophenotypic findings—diffuse S100 positivity, nuclear expression of SOX10, and GFAP decorating fine fibrillary matrix.

Histopathological examination (Figure 3) demonstrated a well-circumscribed spindle-cell tumor without atypia and mitotic activity. Immunohistochemical expression of S100, SOX10, and GFAP was consistent.

The lesion was completely excised, and no early recurrence was observed, consistent with the favorable prognosis reported in the literature [8,9].

Informed consent was obtained from all participants prior to inclusion in the study.

## 3. Discussion

Peripheral nerve sheath tumors (PNSTs) of the oral cavity are rare and may present significant diagnostic challenges due to their variable clinical presentation and biological behavior [5,17]. The two cases presented here illustrate the spectrum of these tumors, ranging from benign schwannoma to malignant peripheral nerve sheath tumor (MPNST), and highlight the potential for clinical overlap.

Oral PNSTs may arise sporadically or in association with hereditary tumor syndromes, particularly NF1 and schwannomatosis. This distinction is clinically important because syndromic tumors may demonstrate different biological behavior, increased malignant potential, and more complex surgical management. NF1-associated MPNSTs are commonly linked to malignant transformation of plexiform neurofibromas and tend to demonstrate more aggressive biological behavior and poorer prognosis compared with sporadic lesions [5,6,7]. Therefore, distinguishing NF1-associated from sporadic MPNSTs is clinically important for diagnostic assessment and patient management.

The differential diagnosis of spindle-cell lesions in the oral cavity includes neurofibroma, fibrosarcoma, leiomyosarcoma, and spindle-cell carcinoma [14]. As seen in the present cases, clinical findings alone are often insufficient for accurate diagnosis, particularly in small or superficially located lesions [12]. This underlines the importance of histopathological and immunohistochemical evaluation.

Histopathological interpretation of PNSTs may be particularly challenging in limited biopsy samples. Early MPNST may partially retain morphological and immunophenotypic features of benign neurofibroma, leading to potential underdiagnosis. NF1-associated tumors frequently demonstrate increased cellularity, hyperchromasia, elevated mitotic activity, increased Ki-67 index, and accumulation of additional genomic alterations involving TP53, CDKN2A, and PRC2-associated genes such as SUZ12 and EED [2,3]. In contrast, schwannomas usually retain diffuse S100 and SOX10 positivity with minimal atypia and low proliferative activity [8,9,15].

Previous studies have shown that oral MPNSTs typically present as enlarging masses with aggressive features, including local invasion and recurrence, although their presentation may be nonspecific and overlap with benign lesions [18]. In addition, neural tumors in the oral cavity may be misinterpreted, particularly in limited biopsy samples [19]. In contrast, schwannomas are generally described as slow-growing, well-circumscribed lesions with a favorable prognosis following complete excision [20].

The findings in our cases are in line with these observations. However, Case 1 raises an additional consideration. The occurrence of a high-grade MPNST at the site of a previously excised lesion initially diagnosed as benign may reflect malignant transformation. The initiated revision confirmed the benign nature of the previously biopsied lesion but elucidated the exact origin of the tumor with implications for clinical management and follow-up.

In the present case, the plexiform architecture and involvement of the mental nerve raised concern for malignant transformation arising from a pre-existing plexiform neurofibroma, a finding considered highly characteristic of NF1-associated MPNST. Although NF1 could not be genetically confirmed, the observed clinicopathological pattern supports this possibility.

In Case 1, additional features supporting malignancy included progressive growth, mental nerve involvement, and markedly elevated proliferative activity [4,17]. Recognition of these clinical and histopathological findings is important because early malignant peripheral nerve sheath tumors may clinically resemble benign lesions. At the molecular level, MPNST development is associated with alterations in tumor suppressor genes such as NF1, TP53, and CDKN2A [3,6].

From a clinical perspective, certain features may raise suspicion for malignancy in peripheral nerve sheath tumors of the oral cavity. These include progressive growth, recurrence after prior excision, pain or paresthesia, and signs of nerve involvement. In the present case, the combination of lesion progression, mental nerve thickening, and elevated Ki-67 supported a malignant process.

In contrast, Case 2 showed features typical of benign schwannoma, including small size, well-defined margins, and indolent behavior [8,9]. Schwannomas are usually encapsulated and associated with a low risk of recurrence after complete excision [8].

Histopathological and immunohistochemical evaluation remains essential for diagnosis [2,14]. Schwannomas typically demonstrate strong S100 expression and low mitotic activity, whereas MPNSTs show increased cellularity, atypia, and higher proliferative indices [2,14].

Surgical excision with negative margins remains the mainstay of treatment for both benign and malignant PNSTs [15,21]. In MPNST, complete resection is the most important prognostic factor, while adjuvant therapy may be considered depending on tumor characteristics and patient factors [16,21,22]. Prognosis remains limited, with reported 5-year survival rates of 30% to 50% [17,23].

Surgical treatment of oral MPNSTs may be especially demanding due to the need to balance oncological radicality with preservation of oral function and facial aesthetics. In Case 1, radical lower lip excision required immediate reconstruction to restore oral competence and an acceptable cosmetic outcome, emphasizing the importance of multidisciplinary surgical planning in malignant oral PNSTs.

Overall, these cases highlight the importance of correlating clinical, imaging, and histopathological findings in the evaluation of peripheral nerve sheath tumors. Comparison is given in Table 1.

While limited by the small number of cases, this report draws attention to a potential diagnostic challenge in recurrent or previously treated lesions, where the distinction between benign and malignant processes may not be straightforward. Careful interpretation and appropriate follow-up should be considered in such situations.

Limitations of the present report include the absence of molecular and genetic testing for NF1-associated alterations, which limited definitive classification of the malignant lesion as syndromic or sporadic. Long-term follow-up data were also limited. In addition, the small number of cases restricts generalizability. Nevertheless, the contrasting biological behavior and histopathological features observed in the present cases contribute to the limited literature regarding oral PNSTs and their diagnostic complexity.

## 4. Conclusions

This two-case series highlights the broad biological spectrum of oral peripheral nerve sheath tumors, ranging from benign schwannoma to aggressive malignant peripheral nerve sheath tumor. Despite partially overlapping clinical presentation, these lesions differ substantially in biological behavior, prognosis, and therapeutic requirements.

Particular attention should be given to recurrent lesions, plexiform architecture, nerve involvement, and increased proliferative activity, as these findings may indicate malignant transformation and possible association with NF1. Distinguishing sporadic from syndromic PNSTs is clinically important because NF1-associated tumors demonstrate increased malignant potential and poorer prognosis.

Histopathological and immunohistochemical evaluation remain essential for accurate diagnosis, although interpretation may be difficult in limited biopsy specimens. Complete surgical excision with negative margins remains the cornerstone of treatment, particularly in malignant tumors where multidisciplinary management and long-term surveillance are critical.

Although limited by the absence of molecular testing and the small number of cases, the present report contributes to the growing literature on rare oral PNSTs and emphasizes the importance of integrating clinical, pathological, and genetic considerations in their evaluation.

## Figures and Tables

**Table 1:** Comparison of clinical and pathological features between the two cases.

Feature	Case 1 (MPNST)	Case 2 (Schwannoma)
Age	76	20
Location	Lower lip	Tongue
Behavior	Malignant	Benign
Growth	Progressive	Intermittent
Nerve involvement	Present	Absent
Ki-67	High (45%)	Low (1%)
Treatment	Radical excision	Local excision
Outcome	Surveillance	Curative

## Data Availability

The data that support the findings of this study are available on request from the corresponding author. The data are not publicly available due to privacy and ethical restrictions.

## Abbreviations

The following abbreviations are used in this manuscript:

CT	Computed Tomography
CD34	Cluster of Differentiation 34
GFAP	Glial Fibrillary Acidic Protein
HPF	High Power Field
IHC	Immunohistochemistry
Ki-67	Proliferation Index Marker (Ki-67 antigen)
MPNST	Malignant Peripheral Nerve Sheath Tumor
MRI	Magnetic Resonance Imaging
NF1	Neurofibromatosis Type 1
NCCN	National Comprehensive Cancer Network
PNST	Peripheral Nerve Sheath Tumor
SOX10	SRY-Box Transcription Factor 10
S100	S100 Protein
